# Case series of aortoenteric fistulas: a rare cause of gastrointestinal bleeding

**DOI:** 10.1186/s12876-021-01629-4

**Published:** 2021-02-02

**Authors:** Jia Luo, Wen Tang, Mengran Wang, Yao Xiao, Manhong Tan, Chunyan Jiang

**Affiliations:** Department of Internal Medicine and Geriatrics, Beijing Friendship Hospital, Capital Medical University, No. 95, Yong’ an Road, Xicheng District, Beijing, 100050 People’s Republic of China

**Keywords:** Aortoenteric fistula, Gastrointestinal bleeding, Clinical characteristics, Management

## Abstract

**Background:**

Aortoenteric fistula (AEF) is a rare cause of gastrointestinal bleeding and is often misdiagnosed in clinical practice. Herein, a case series of AEFs are presented and the clinical characteristics, diagnosis, and management strategies are summarized.

**Methods:**

A retrospective analysis was performed on consecutive hospitalized patients with a final diagnosis of AEF at Beijing Friendship Hospital, Capital Medical University, between January 1, 2007 and March 31, 2020. The clinical data including diagnostic and management procedures as well as outcomes were collected and summarized.

**Results:**

A total of nine patients were included in this study, five with primary AEF and four with secondary AEF. Eight of the patients were male, and the median age was 63 years. The fistulas were located in both the small intestine and the colon. All patients presented with gastrointestinal bleeding and pain, followed by weight loss, anorexia, and fever. A typical abdominal triad was found in only two cases. Seven patients experienced complications with preoperative abdominal infections and sepsis, and multiple organ failure occurred in four of these patients. All patients were assessed by computed tomography and five underwent abdominal and/or iliac aorta angiography. Two of these patients showed contrast agent leakage from the abdominal aorta into the intestine. Two cases were diagnosed with AEF by endoscopy before the operation. Eight patients received surgery and six patients survived.

**Conclusions:**

AEF is a rare cause of gastrointestinal bleeding that is associated with high mortality. Gastrointestinal bleeding and pain are the most common presentations. Timely diagnosis and multidisciplinary management are crucial to achieve a positive outcome.

## Background

Aortoenteric fistulas (AEFs) are a rare cause of gastrointestinal bleeding [1]. They were first reported in the early nineteenth century and were defined as the presence of abnormal channels between the aorta and the gastrointestinal tract [2]. AEF is a complex and devastating clinical condition that leads to gastrointestinal bleeding and/or severe sepsis, and it can be classified into two categories: primary aortoenteric fistula (PAEF), and secondary aortoenteric fistula (SAEF) [3–5]. PAEF is defined as a spontaneous communication between the native aorta and any portion of the gastrointestinal tract, and it is most often the result of compression against an abdominal aortic aneurysm (AAA). SAEF is a complication of an AAA repair that occurs when there is a false communication between an enteric structure and a previous aortic graft. The prevalence of PAEF varies depending on the study base. Voorhoeve and colleagues have described a prevalence of 0.04–0.07% among patients who died due to a massive GI hemorrhage [6], but it is higher in patients with AAAs and ranges from 0.69 to 2.36% [7, 8]. The incidence of SAEF has been reported to range from 0.36 to 1.6% [9, 10].

Owing to the nonspecific and subtle clinical presentations, the diagnosis of AEF is difficult. Meanwhile, AEF carries an extremely high mortality rate (almost 100%) if left untreated [11]. In this article, we reviewed all AEF patients admitted from Jan 2007 to March 2020 at a single institution, analyzed their clinical characteristics, methods used for their diagnosis, management modalities, and outcomes, to help clinicians better their recognition of this uncommon disease to improve its diagnosis and management.

## Methods

This was a retrospective observational study. Consecutive hospitalized patients with gastrointestinal bleeding and a final diagnosis of AEF in Beijing Friendship Hospital, Capital Medical University from January 1, 2007 to March 31, 2020, were reviewed. The medical ethical committee of Beijing Friendship Hospital, Capital Medical University approved the study protocol and declared the study as not covered under WMO (Medical Research Involving Human Subjects Act) and therefore informed consent was not required. Data regarding demographics, clinical manifestations, physical examinations, laboratory data, imaging, treatments, and outcomes were collected and analyzed. Diagnosis of AEF was confirmed by endoscopy, radiography, and/or surgical findings.

## Results

### Demographics

Through the medical record system, approximately 3,000 inpatients with a major diagnosis of gastrointestinal bleeding in our hospital were retrospectively reviewed from January 1, 2007 to March 31, 2020. A total of nine patients with the final diagnosis of AEF were included in the analysis (Table 1). There were eight men and one woman, with a median age of 63 years (range 21–86 years). Five patients had concomitant diseases including hypertension, coronary heart disease, diabetes mellitus, rheumatic disease, and lymphoma. Five were diagnosed as PAEF, and the remaining four had SAEF resulting from a previous surgery to repair an aortic aneurysm. Among the four SAEF patients, the time interval between surgery to initial symptoms of AEF was more than two years in three patients (range 2–5 years), and only one patient (case 7) developed an initial symptom of AEF at 8 months post-surgery. The fistulas were located in the duodenum (cases 1, 3, 5, 7), jejunum or ileum (cases 1, 6, 8, 9), or the colon (case 2, 4).

**Table 1:** Clinical characteristics of patients with AEF

No	Sex/age	Smoking history	Comorbidities	PAEF/SAEF	AEF location (bowel)	Clinical features								
						GI bleeding	Time interval	Fever	Pain location	Accompanying situation	Pulsating mass	Pulsation of lower extremity artery	Sepsis/CI	MOF
1	M/21	−	−	PAEF	Duodenum and jejunum	Hematochezia; hematemesis; syncope; hypovolemic shock	3 days	−	Upper abdomen	Nausea; vomiting; pneumonia; pleural effusion (bilateral)	−	−	+	ARDS, AKI, MI, ALI, RM, DIC
2	M/46	4PY	DM	PAEF	Colon	Hematochezia; syncope; hypovolemic shock	5 days	38.2℃	Back	Pneumonia; pleural effusion (bilateral)	−	Decreased	+	−
3	M/61	80PY	AS, RA	PAEF	Duodenum	Hematochezia; syncope; hypovolemic shock	1 month	−	Umbilical, abdominal	Anorexia; weight loss	−	Decreased	−	−
4	M/65	10PY	−	SAEF*	Colon	Hematochezia	3 month	38.8℃	Lower right abdomen	Anorexia; weight loss; pneumonia; pleural effusion (unilateral)	+	−	+	AKI, ALI
5	M/85	7.5PY	HT	SAEF^†^	Duodenum	Melena	5 month	38.7℃	Right suprapubic	Anorexia; Weight loss; pneumonia; pleural effusion (bilateral)	+	Decreased	+	AHF, AKI
6	F/86	−	HT, NHL	SAEF^‡^	Jejunum or ileum	Hematochezia; syncope; hypovolemic shock	7 days	40.5℃	Entire abdomen	Nausea; vomiting; anorexia; weight loss; pneumonia; pleural effusion (bilateral)	−	Decreased	+	AKI; MI
7	M/68	50PY	CHD	SAEF^‡^	Duodenum	Hematochezia	4 month	40℃	Upper abdomen and back	Anorexia; Pleural effusion (bilateral)	−	Decreased	+	−
8	M/74	50PY	−	PAEF	Jejunum or ileum	Hematochezia; hematemesis; syncope; hypovolemic shock	4 days	−	Upper abdomen	Nausea; vomiting	−	−	−	−
9	M/63	30PY	−	PAEF	Jejunum or ileum	Melena; syncope; hypovolemic shock	21 days	−	Lower of right abdomen	Anorexia; weight loss	−	−	+	ALI

*AEF* aortoenteric fistula, *PY* pack years, *DM* diabetes mellitus, *AS* ankylosing spondylitis, *RA* rheumatoid arthritis, *HT* hypertension, *NHL* non-Hodgkin lymphoma, *CHD* coronary heart disease, *PAEF* primary aortic-enteric fistula, *SAEF* secondary-enteric aortic fistula, *CI* celiac infection, *MOF* multiple organ failure, *ARDS* acute respiratory distress syndrome, *AKI* acute kidney injury, *MI* myocardial injury, *ALI* acute liver injury, *RM* rhabdomyolysis, *DIC* disseminated intravascular coagulation, *AHF* acute heart failure

^*^History of iliac aneurysm bypass surgery, ^†^history of abdominal aortic dissection and right iliac aneurysm stenting, ^‡^history of abdominal aortic aneurysm stent grafting

### Clinical presentations and physical examinations

Four of the nine patients had presented with intermittent abdominal pain as the initial symptom (cases 3, 4, 8, 9). The second prominent initial symptom was gastrointestinal bleeding. Melena was seen in cases 5 and 6, and hematochezia developed in case 1. The other two patients presented with fever (case 7) or syncope and hemorrhagic shock (case 2) as the initial symptoms. All patients presented with intermittent gastrointestinal bleeding lasting from three days to over five months, with six patients having experience a duration time longer than a week. The presentations included hematochezia (7/9), hematemesis (2/9), melena (2/9), hemorrhagic shock (6/9), and/or syncope (6/9). All patients experienced pain related to the location of the lesion, such as abdominal pain (7/9), back pain (2/9), or suprapubic pain (1/9). Six patients experienced weight loss and anorexia. Other manifestations included moderate to high fever (5/9), as well as nausea and vomiting (3/9).

Physical examinations revealed a pulsating abdominal mass in two patients, disturbance of consciousness in two patients, abdominal tenderness in seven patients, and decreased pulsation of lower extremity arteries in five patients. The typical triad of abdominal of pain, a pulsating mass and gastrointestinal hemorrhage was observed in only two out of the nine patients (cases 4, 5).

There were seven patients (cases 1, 2, 4, 5, 6, 7, 9) complicated with abdominal infections and sepsis, and six of these patients (cases 1, 2, 5, 6, 7, 9) were positive for pathogens. The pathogenic bacterial and fungal species identified were *Staphylococcus hominis, Staphylococcus hemolyticus, Enterococcus faecalis, Candida albicans*, *Streptococcus gordonii*, *Enterococcus faecium, Candida tropicalis,* and *Streptococcus constellatus*. Except for case 6, which had blood cultures, the rest of the patients had samples cultured from the intestinal fistula and surrounding purulent tissues obtained during the operation. Moreover, six patients (cases 1, 2, 4, 5, 6, 7) were complicated with pulmonary infections and pleural effusion.

Multiple organ failure occurred in four patients (cases 1, 4, 5, 6). The manifestations of organ failure included hypovolemic shock (cases 1, 2, 3, 6), acute kidney injury (cases 1, 4, 5, 6), heart failure or myocardial injury (cases 1, 5, 6), liver function injury (cases 1, 4), acute respiratory distress syndrome (case 1), rhabdomyolysis (case 1), and disseminated intravascular coagulation (case 1).

### Laboratory data and imaging findings

In laboratory testing (Table 2), four of the nine patients had leukocytosis, while the percentage of neutrophils was increased in all patients. Serum C-reactive protein (CRP) levels were elevated in seven patients, and procalcitonin (PCT) levels increased were increased in five patients. Anemia occurred in all patients, with a median hemoglobin level of 7.3 g/dL (range 4.8–9.6 g/dL). Eight patients experienced moderate to severe hypoalbuminemia, with a median albumin level of 25.8 g/L (range 13.6–35.7 g/L).

**Table 2:** Laboratory testing and imaging results, surgery and outcomes

No	WBC (× 10^9^/L)	HGB (g/dL)	CRP (mg/L)	PCT (ng/mL)	ALB (g/L)	EGD	Colonoscopy	Arteriography	Bacterial culture	Surgery	Outcome
1	5.10	6.90	36.00	> 200	13.6	*	-	^†^	*Staphylococcus hominis*	Endovascular stent graft	Died
2	4.90	4.80	66.00	< 0.05	29.6	Chronic gastritis	NA	^†^	*Staphylococcus haemolyticus*	Endovascular stent graft + extraanatomical bypass grafting^‡^	Discharged
3	6.71	9.60	5.80	0.21`	35.7	Chronic gastritis	-	-	NA	Endovascular stent graft	Discharged
4	18.01	8.80	79.00	0.53	21.7	Chronic gastritis	NA	NA	-	EL	Died
5	8.13	5.82	118.00	0.26	33.0	Chronic gastritis	-	NA	*Enterococcus faecalis* + *Candida albicans*	Extraanatomical bypass grafting	Discharged
6	20.40	6.02	28.02	13.25	24.1	NA	NA	NA	*Streptococcus gordonii*	Refused	Died
7	17.15	8.60	56.00	0.56	18.5	Chronic gastritis, Reflux esophagitis	-	NA	*Enterococcus faecium* + *Candida tropicalis*	Extraanatomical bypass grafting	Discharged
8	7.25	8.80	8.00	0.06	31.1	NA	NA	-	-	Endovascular stent graft	Discharged
9	17.62	6.50	98.00	54.31	24.8	Chronic gastritis	-	-	*Streptococcus constellatus*	Endovascular stent graft + Extraanatomical bypass grafting^‡^	Discharged

*WBC* white blood cell count, *HGB* hemoglobin, *CRP* C-reactive protein, *PCT* procalcitonin, *ALB* albumin, *EGD* esophagogastroduodenoscopy, *EL* exploratory laparotomy, *NA* non-applicable

^*^Gastric ulcer at stage A2, duodenal horizontal mucosa uplift with apical thrombus, - negative findings, ^†^extravasation of contrast medium into the intestine, ^‡^first step, endovascular stent graft in an emergency, second step, extra-anatomic reconstruction by artery bypass grafting

**Fig. 1:**
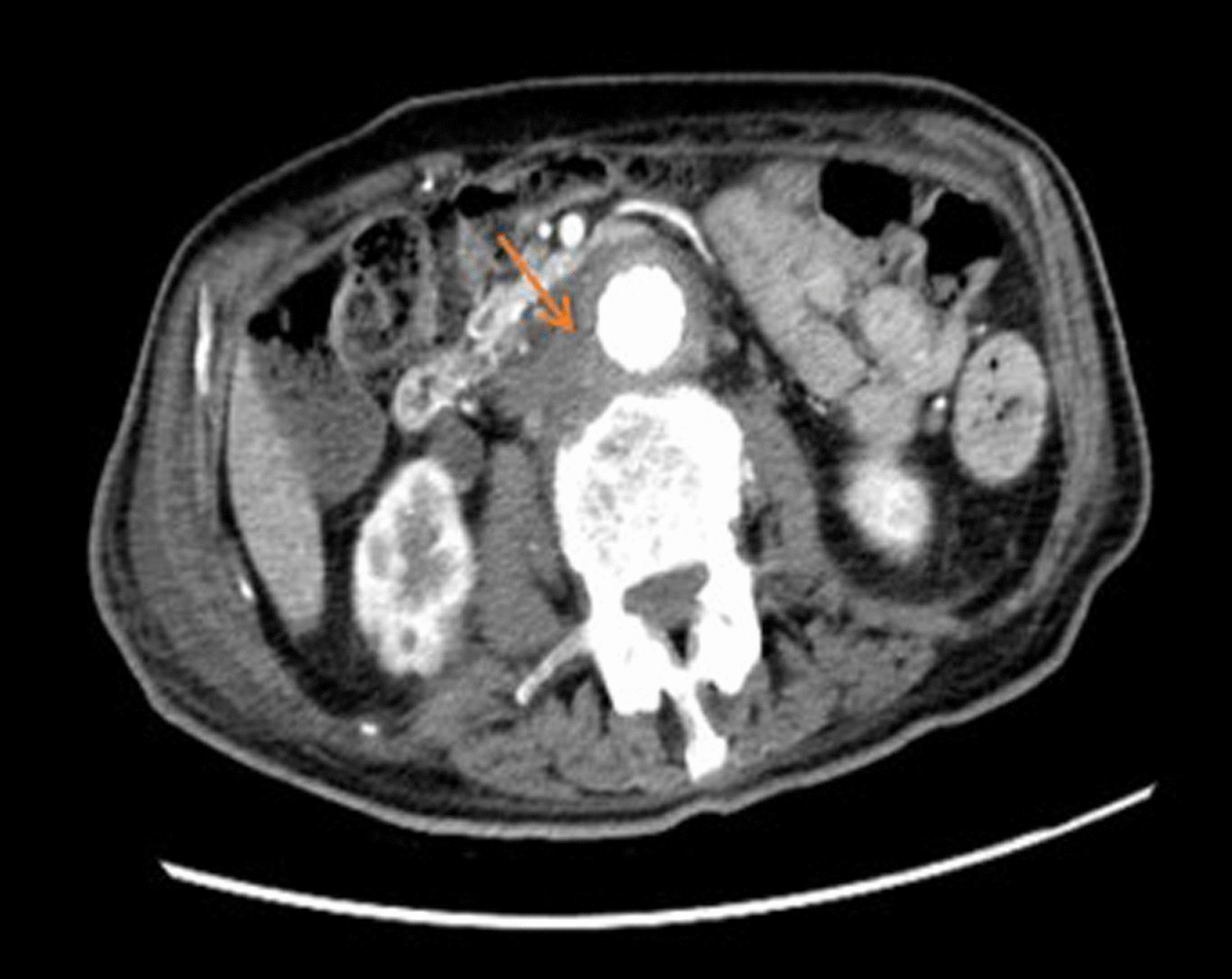
Computed tomography (CT) showed equi- or low-density soft tissue collection around the aorta and an unclear boundary between the aorta and bowel

**Fig. 2:**
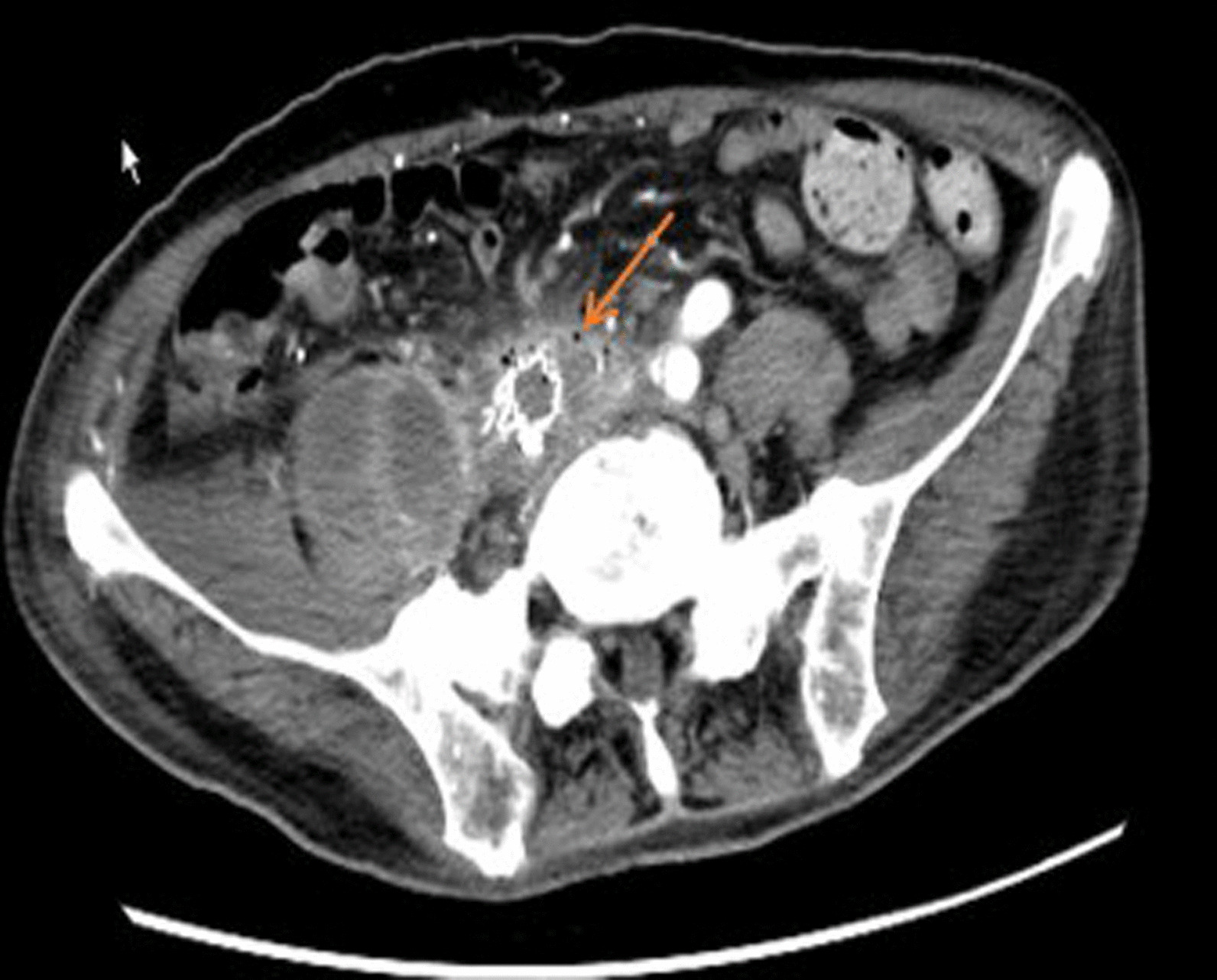
Computed tomography (CT) showed ectopic gas within or adjacent to the right external iliac artery, and equi- or low-density soft tissue collection around the right external iliac artery as well as encapsulated fluid around the right psoas major and iliacus muscles

All patients underwent computed tomography (CT) imaging, and the findings from the CT scan were as follows (Table 3): effacement or blurred borders of fatty planes around arteries (7/9 cases); soft tissue collection around the aorta > 5 mm (7/9 cases; Fig. 1); ectopic gas within or adjacent to the aorta (4/9 cases; Fig. 2); intravenous contrast within the gastrointestinal lumen or around the aorta (3/9 cases); calcification of the arterial wall (5/9 cases); local abdominal aortic ulcers (2/9 cases); intestinal wall thickening or compression (4/9 cases); hydroureteral dilatation (2/9 cases); and peri-renal fascia thickening (2/9 cases). Five patients underwent abdominal and/or iliac aorta angiography, and in two patients (cases 1, 2) contrast agent leakage from the abdominal aorta into the intestine was detected. Angiography in case 3 revealed abdominal aortic aneurysm dilatation at the middle segment, but no contrast agent was detected flowing into the intestine.

**Table 3:** Findings from CT images of patients with AEF

	CT images	No. of cases
A	Effacement or blurred borders of fatty planes around arteries	Case 1, 2, 3, 5, 6, 7, 9
B	Soft tissue collection around the aorta > 5 mm	Case 1, 3, 4, 5, 6, 7, 9
C	Ectopic gas within or adjacent to the aorta	Case 1, 4, 5, 7
D	Intravenous contrast within the GI lumen or around the aorta	Case 1, 6, 9
E	Calcification of the arterial wall	Case 2, 4, 5, 8, 9
F	Local abdominal aortic ulcers	Case 4, 5
G	Intestinal wall thickening or compression	Case 2, 4, 6, 9
H	Hydroureteral dilatation	Case 4, 5
I	Peri-renal fascia thickening	Case 4, 6

*CT* computed tomography, *AEF* aortoenteric fistula, *GI* gastrointestinal

### Gastrointestinal endoscopy

**Fig. 3:**
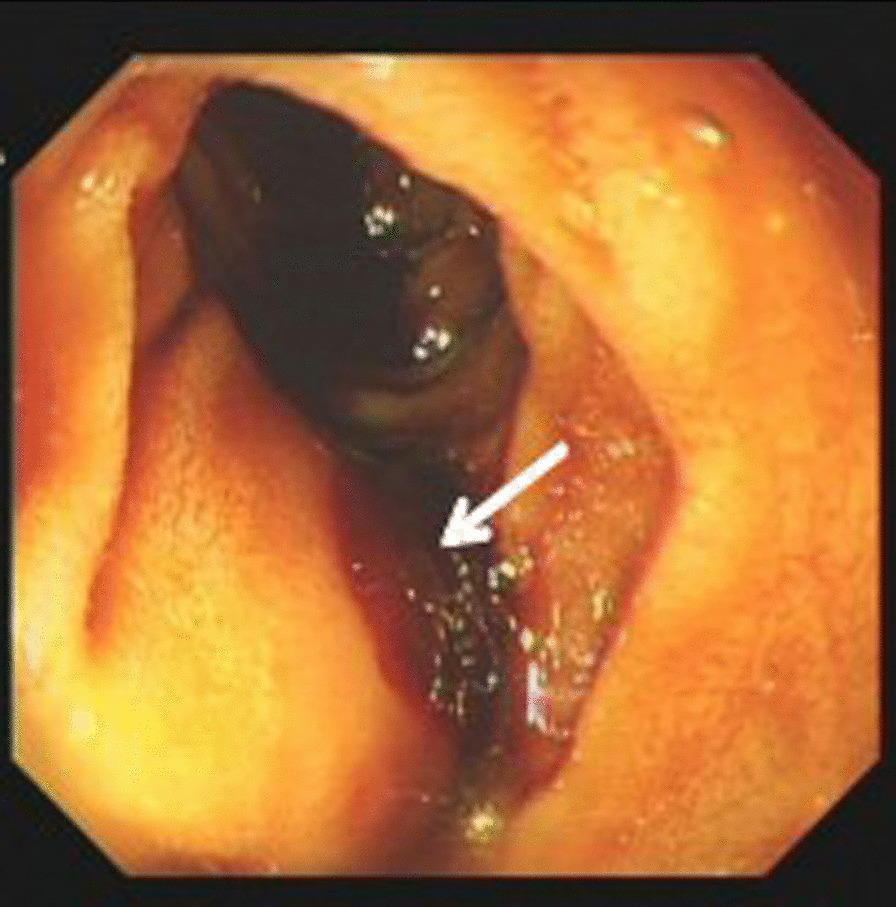
Duodenoscopy showed a mucosa uplift at the beginning of the horizontal section of the duodenum indicating external pressure from the aneurysm, with an apical thrombus suggesting the presence of recent bleeding

**Fig. 4:**
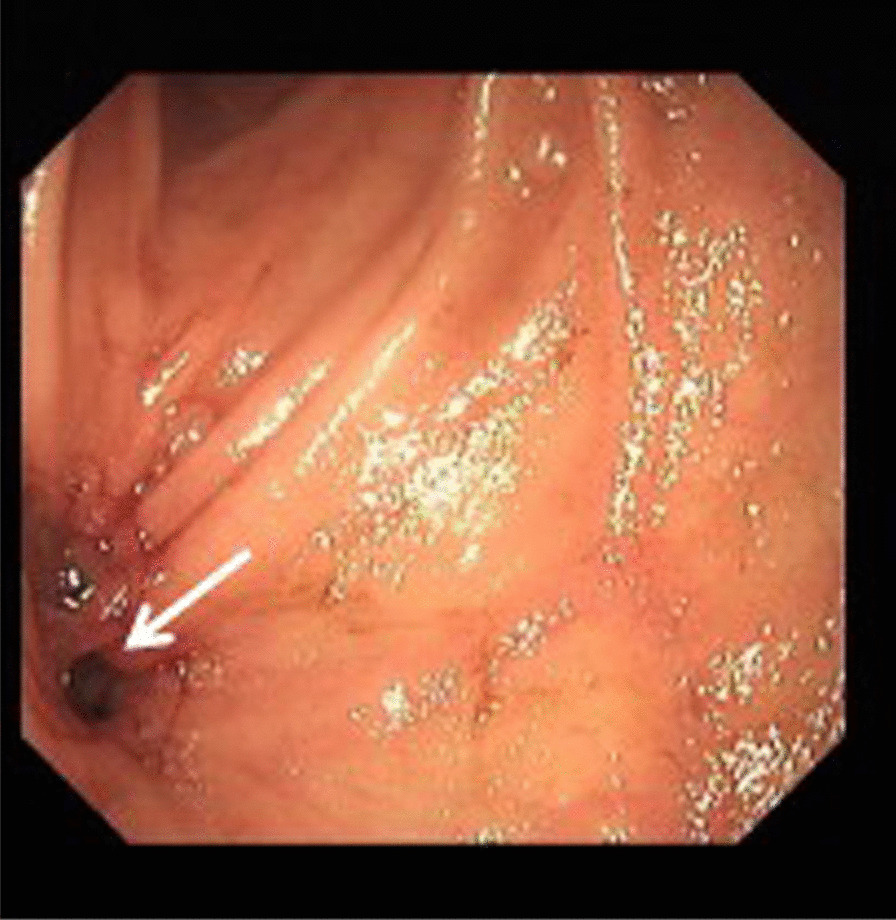
Duodenal endoscopy showed a fistula in the horizontal segment of the duodenum, where the aortic graft had penetrated the bowel wall

To identify the cause of gastrointestinal bleeding, seven patients underwent a total of nine esophagogastroduodenoscopys (EGD), and six patients had negative endoscopy findings. Two patients (cases 3, 5) underwent two EGD examinations each. Only case 1 had a reported gastric ulcer at stage A2 [12], with a mucosa uplift at the initial horizontal section of the duodenum indicating the external pressure of an aneurysm, with an apical thrombus suggesting the presence of recent bleeding (Fig. 3). Case 5 initially underwent a gastroscopy, and the result suggested chronic gastritis, but CT findings strongly implicated the possibility of an aortic-intestinal fistula, which was subsequently confirmed by duodenoscopy (Fig. 4). Five patients (cases 1, 3, 5, 7, 9) underwent colonoscopy examinations, and the results were unremarkable.

### Treatments and outcomes

All patients underwent emergency treatments including the prohibition of drinking and eating, resuscitation, hemostasis, transfusion of packed red blood cells, use anti-infection agents, etc. All patients were transferred to the operating theatre except for an 86-year-old woman (case 6 diagnosed as SAEF), who gave up surgical exploration and died quickly within one week.

Two cases of PAEF (cases 3, 8) without celiac infection underwent endovascular approaches for the placement of an abdominal aorta stent-graft, and the cause of PAEF was identified as AAA. Two other cases of PAEF (cases 2, 9) had unstable clinical signs and celiac infections. Therefore, decisions were made to place an emergency temporary artery stent-graft first, and then to perform a partial intestinal resection, end-to-end anastomosis, abdominal abscess resolution and drainage, retroperitoneal mass resection, artery stent removal, artery ligation, and femoral-femoral artery bypass grafting. Histological examination revealed necrotizing inflammation in the aortic wall at the location of the fistula, a chronic ulcer in the intestinal wall with inflammatory exudates on the surface, and retroperitoneal microabscesses. All four of these PAEF patients survived their respective operations. Case 1, the fifth PAEF patient, underwent an emergency abdominal aorta endovascular stent-graft, and died 40 days after the surgical intervention due to uncontrolled bleeding, sepsis, and multiple organ failure during the perioperative period.

Three cases of SAEF (cases 4, 5, 7) underwent surgical treatment. Case 4 had an emergency exploratory laparotomy performed and died three days after the operation due to uncontrolled bleeding, sepsis, and multiple organ failure during the perioperative period. Case 7 underwent an extra-anatomic bypass graft reconstruction surgery and had an uneventful perioperative period. Case 5 underwent an exploratory laparotomy, extra-anatomic left axillofemoral bypass graft reconstruction, aortic stent graft excision, infrarenal abdominal aortic suture, left common iliac artery ligation, extensive surgical debridement, and retroperitoneal abscess resolution and drainage, along with duodenal defect repair and jejunal feeding tube placement. After such a complicated surgery with several life-threatening complications, he survived with multidisciplinary team management.

## Discussion

AEF is a rare yet lethal cause of gastrointestinal bleeding, and two types of AEF are recognized: PAEF and SAEF. A PAEF occurs in the setting of an unrepaired aortic aneurysm sac that erodes into the gastrointestinal lumen in the presence of predisposing factors. Most commonly, these factors include atherosclerosis (60–85%) and infection (15%), and more rarely are a result of malignant tumors, duodenal ulcers, foreign materials or complications after radiotherapy [13, 14]. SAEF often occurs after aortic reconstruction of aneurysms or occlusive diseases, in which the artificial blood vessels or sutures used become corroded and protrude into the digestive tract [9, 10]. The incidence of SAEF as a complication of repair and reconstruction is increasing with the increasing number of revascularizations for aortic aneurysm and severe aortoiliac atherosclerosis. AEF is more common in males, paralleling the incidence of AAA and aortic surgery. The male to female ratio is 3:1 for PAEF and 8:1 for SAEF [15]. In our study, eight of the nine AEF patients were men, and seven of them had a long history of smoking.

AEF is a devastating diagnosis regardless of its etiology, with bleeding and sepsis as the most common initial presentations [16, 17]. Bleeding episodes range from a minor hemorrhage (such as a herald bleed) to massive, life-threatening bleeding or overt rapid exsanguination [16, 18, 19]. A herald bleed is a transient or self-limiting early hemorrhage prior to the occurrence of a life-threatening hemorrhage hours to days later, and they appear in approximately 20–75% of cases [18, 19]. In our analysis, four patients initially presented with massive bleeding and hemorrhagic shock, while the other five cases presented with recurrent melena or hematochezia for one to three months. Interestingly, although abdominal pain had been reported in only 35% of patients [20, 21], nearly all of our patients experienced pain, which manifested as abdominal, lower back, or even suprapubic pain, depending on the location and extent of the lesion. Therefore, it can be reasonably suggested that pain as an alarming symptom should be taken seriously by clinicians. The classical triad (gastrointestinal bleeding, pain, and a pulsating mass) has been proposed for the diagnosis of AEF. However, it is not a frequent clinical presentation in actual clinical practices. Saers and Scheltinga have published that the classical triad was present in only 11% of 81 patients [22], and consistently, only two of the nine patients presented with a typical triad in our study. Our study suggested that the lower occurrence of the classical triad may be associated with the subtle clinical presentation of the pulsating mass. Except for fatal gastrointestinal bleeding, both PAEF (3/5) and SAEF (4/4) patients were prone to present with fever and sepsis, which directly affects the subsequent treatment and prognosis. In particular, AEF patients with intermittent fever or septicemia are often misdiagnosed with other infections, such as pneumonia [23], especially in cases where early bleeding is not substantial. Other symptoms of AEF include malaise, weight loss, graft thrombus with lower extremity ischemia, and other nonspecific symptoms.

A delay in the diagnosis can be particularly disastrous in the setting of AEF, but it is difficult to diagnose prior to an operation. As such, the preoperative diagnosis rate is only 14.3–36% [24]. CT and EGD are most frequently used to diagnose AEF, with an abdominal contrast-enhanced CT scan being the preferred initial diagnostic examination of choice. Unfortunately, none of the diagnostic modalities are perfect for the diagnosis of AEF. A retrospective study showed that CT angiography can identify a fistula in 35% of cases, whereas an EGD can only identify a fistula in 25% of cases [25]. In contrast, exploratory laparotomies had the highest sensitivity and specificity (91–100%) [26].

CT scans can be used to locate aortoenteric fistulas and determine whether there is an infection or abscess in the abdomen [27]. This method is reliable, widely available, quick to perform, and relatively easy to interpret, especially in patients who are hemodynamically unstable and if the physician has a high suspicion of AEF. The most important CT finding in AEF is ectopic gas either within or directly adjacent to the aortic lumen or the graft [28]. However, in a series analysis, bowel wall thickening, effacement of the periaortic fat plane, and periaortic soft tissue/fluid were seen in virtually every patient, while ectopic gas was seen in only 56% patients [29]. The extravasation of contrast from the aorta into the bowel lumen is a highly specific sign, but extremely rare [30]. According to our study, the most common CT findings in cases of aortoenteric fistulas were effacement or blurred borders of fatty planes around arteries and soft tissue collection around the aorta > 5 mm. Ectopic gas within or around the graft or aorta manifested in only four of the nine cases.

Endoscopy is the most useful investigation to determine the origin of gastrointestinal bleeding, but the accuracy for AEF diagnosis is only 25–40% [11, 22, 31, 32]. Peck et al*.* [24] reported that 29% of the cases (8/28) were diagnosed as AEF by endoscopy, but 32% of patients (9/28) were misdiagnosed by endoscopy. Considerable variation may exist in the diagnostic yield of EGD, which may partly be explained by operative technique or experience. The low sensitivity and specificity may be related to the following factors: (1) most AEFs occur in the third or the fourth part of the duodenum and gastroscopy usually only reaches the second segment of the duodenum, so the third and fourth segments are easily missed; (2) in the case of gastrointestinal bleeding, there may be a large amount of old and new blood that has accumulated in the gastrointestinal tract, resulting in unclear endoscopic fields of vision, which affects observation; (3) endoscopists were prone to misdiagnose the endoscopic findings of AEF as ulcers, polyps, erosion, and other coexisting symptoms, thus leading to a missed opportunity at the most crucial time-point for correct management; and (4) AEF patients with unstable hemodynamic status found the endoscopic examination difficult to tolerate. Therefore, some experts suggested that an enteroscope or pediatric colonoscope should be utilized instead of a regular gastroscope to aid in better visualization of the distal duodenum and proximal jejunum [33, 34]. Positive findings suggestive of AEF on EGD include visible graft, bleeding, adherent blood clots, an ulcer, or a pulsatile mass [9, 26, 32].

In both PAEF and SAEF, the duodenum is the portion of the gastrointestinal tract most frequently involved, especially in the third section where the duodenum is in the closest proximity to the aorta [18, 26, 35, 36]. Kakkos et al. [17] noted that the most common location of AEF is the duodenum (62% of all cases and 77.6% [510/657] of those reporting the exact AEF location[s]), followed by other sections of the small intestine as well as the colon, and cases with two fistulas are rare. In our study, fistulas were located in the duodenum (4/9), other areas of small intestine (4/9) and colon (2/9). One patient (case 1) was confirmed during surgery to have fistulas in both the duodenum and jejunum.

The optimal outcome of AEF requires confirmation of the diagnosis, quick control of the hemorrhage, repair of the bowel defect, eradication of associated infection, and revascularization. The only curative treatment for AEF is surgery, without which the mortality approaches 100%. There are multiple surgical strategies to maintain perfusion while eradicating the infection and restoring intestinal continuity. The methods of repair for primary and secondary AEF are without significant differences. Procedures are loosely grouped into in situ reconstructions and extra-anatomic bypass with aortic ligation [37]. The latter represents the historical gold standard. In recent decades, however, in situ methods of maintaining arterial perfusion have gained popularity, in light of the modest outcomes following axillobifemoral bypass and aortic stump ligation. A review and pooled data analysis of 823 patients indicated that endovascular surgery is associated with better early survival than open surgery for SAEFs, especially in those with significant bleeding [17]. However, this difference diminishes with time as the two-year survival is 40% for open surgery and 51% for an endovascular approach.

Sepsis remains a life-threatening complication of AEF, and failure to control it may result in mortality rates of 60% [38]. In a systematic review, preoperative infection [OR 9.36 (2.24–39.12)] was identified as a predictor of adverse outcomes in the univariate analysis, and patients in the infection group (persistent or recurrent) had a worse 30-day mortality rate when compared to patients without infection (16.67% *vs* 0.00%, *p* = 0.042) [38]. Given the complicated surgical course and proximity of the intestinal microbiota, the early administration of broad-spectrum antibiotics should target gram-positive and gram-negative organisms as well as anaerobes [23]. Seven patients in our cohort had abdominal infections and septicemia. Gram-positive cocci (*Staphylococcus, Enterococcus*, *Streptococcus*) were the main pathogenic microorganisms in the culture, and two patients also had fungal infections (*Candida*). The CRP and PCT may help guide the duration of antibiotics and monitor the response of therapy. It should be noted that six out of nine patients developed pulmonary infection and pleural effusion, which may be associated with hypoproteinemia or immunocompromised conditions. Other life-threatening complications included multiorgan failure, acute coronary events, limb ischemia leading to gangrene or amputation, liver failure, and acute respiratory failure [23]. The incidence of multiple organ failure in our study was 4/9 (44.44%), and it increased to 3/5 (60%) if the patients were elderly (aged ≥ 65 years), since they were more prone to develop acute kidney injury.

## Conclusion

In conclusion, AEF is one of the rare causes of gastrointestinal bleeding that can be fatal if not diagnosed and treated in a timely manner. Delays persist due to a lack of knowledge regarding the patient’s medical history, common clinical presentations, as well as the appropriate role of diagnostic tools. Clinicians should highly suspect a diagnosis of AEF if a patient presents with massive gastrointestinal hemorrhage, hypovolemic shock, various pains, and septicemia, especially in middle-aged or older men with a history of heavy smoking. The abdominal contrast-enhanced CT scan should be the first-line imaging modality for the detection of aortoenteric fistulas, since endoscopy has a limited value for diagnosis. All clinicians should be very aware that the third segment of the duodenum is the most frequently reported location of AEF. Multidisciplinary management is crucial and is comprised of urgent exploratory surgical intervention and antibiotics, as well as multiorgan function support.

## Data Availability

The datasets used and/or analyzed during the current study are available from the corresponding author on reasonable request.

## Abbreviations

AEF: Aortoenteric fistula
PAEF: Primary aortoenteric fistula
SAEF: Secondary aortoenteric fistula
AAA: Abdominal aortic aneurysm
CRP: C-reactive protein
PCT: Procalcitonin
CT: Computed tomography
EGD: Esophagogastroduodenoscopy
